# Reorganization of Protein Tyrosine Nitration Pattern Indicates the Relative Tolerance of *Brassica napus* (L.) over *Helianthus annuus* (L.) to Combined Heavy Metal Treatment

**DOI:** 10.3390/plants9070902

**Published:** 2020-07-16

**Authors:** Gábor Feigl, Ádám Czifra, Árpád Molnár, Attila Bodor, Etelka Kovács, Katalin Perei, Vivian Jebet, Zsuzsanna Kolbert

**Affiliations:** 1 Department of Plant Biology, University of Szeged, Közép Fasor 52, H6726 Szeged, Hungary; czifra.adam1@gmail.com (Á.C.); molnara@bio.u-szeged.hu (Á.M.); vivianjebet@gmail.com (V.J.); kolzsu@bio.u-szeged.hu (Z.K.); 2 Department of Biotechnology, University of Szeged, Közép Fasor 52, H6726 Szeged, Hungary; bodora@bio.u-szeged.hu (A.B.); kovacset@bio.u-szeged.hu (E.K.); perei@szbk.hu (K.P.); 3 Institute of Environmental and Technological Sciences, University of Szeged, Közép Fasor 52, H6726 Szeged, Hungary; 4 Institute of Biophysics, Biological Research Centre, Temesvári Körút 62, H6726 Szeged, Hungary

**Keywords:** heavy metals, nitric oxide, tyrosine nitration, sunflower, rapeseed

## Abstract

Metal-polluted areas, especially where municipal sewage is used as fertilizer, often have high concentrations of more than one metal. The development of the root system is regulated by a complex signaling network, which includes reactive oxygen and nitrogen species. The delicate balance of the endogenous signal system can be affected by various environmental stimuli including heavy metals (HMs) in excess. Our goal was to analyze the microelement homeostasis, root architecture, and to determine the underlying changes in the nitro-oxidative status in the root system of rapeseed (*Brassica napus* L.) and sunflower (*Helianthus annuus* L.) subjected to combined HM treatments. The effect of model-sewage in two different layouts was simulated in rhizotron system by only supplementing the highest HM concentrations (Cd, Cr, Cu, Hg, Ni, Pb, and Zn) legally allowed. The two species reacted differently to combined HM treatment; compared to the relatively sensitive sunflower, rapeseed showed better metal translocation capability and root growth even at the more severe treatment, where the pattern of protein tyrosine nitration was reorganized. The obtained results, especially the increased nitric oxide content and changed pattern of tyrosine nitration in rapeseed, can indicate acclimation and species-specific nitro-oxidative responses to combined HM stress.

## 1. Introduction

Heavy metal (HM) contamination of soils and waters is an emerging challenge for the environment and agriculture. Particularly in the developing countries, where HM contaminated wastewaters are discharged to the environment in an increasing manner due to the fast development of industries. Since the availability of clean freshwater is becoming increasingly limited, more and more places use wastewater for irrigation. In addition to containing valuable macro- and micronutrients, which in optimal concentration might be beneficial for plant growth and development [1], domestic wastewater might also contain a considerable amount of non-biodegradable HMs, which in excess, are able to accumulate in the food chain, potentially causing toxicity symptoms [2,3]. Among the many possible contaminants, cadmium (Cd), chromium (Cr), copper (Cu), lead (Pb), mercury (Hg), nickel (Ni), and zinc (Zn) are the most concerning toxic HMs [3], whose properties and effects on plants are discussed in the Supplementary Materials (Table S1).

As being indirectly in contact with the soil, plant roots have an essential role in the uptake and translocation of HMs. Nonetheless, plants try to minimize the adverse effect of HMs by either avoiding or excluding them. Dealing with the fate of HMs, many studies revealed that they affect a wide range of processes, including the disturbance of germination and early development, genotoxicity, disintegration of membranes, inactivating enzymes or even causing oxidative and nitrosative stress [4,5,6]. At low concentrations, HMs are able to trigger a positive adaptation mechanism called stress-induced morphogenic response, which is a special combination of inhibited longitudinal growth of the primary root and induced lateral root development [7], resulting in a shallower but more extensive root system with potentially better tolerance against HMs [4].

The early development of the root system is crucial in the life of a plant. In addition to ensure physical stability for the whole plant, it is responsible for the water and nutrient uptake and also might be practically (e.g., phytoremediation) relevant. The development of the root system is regulated by a complex and a diverse signaling network [8]. In addition to hormonal factors, reactive oxygen and nitrogen species (ROS and RNS) play an important role in the regulation of root development. The gaseous signal molecule nitric oxide (NO) is considered to possess a principle role in the complex signal transduction network behind the whole process [9]. Formation of ROS, including, superoxide anion (O_2_˙^−^), hydroxyl radical (˙OH), and hydrogen peroxide (H_2_O_2_) [10], can be linked to a wide range of stress responses [11]. This suggests an important role as intermediates in metal stress responses, as well as their connections to the signalization of RNS. As a group of molecules, RNS consist of several reaction products of NO, such as the radical nitrogen dioxide, the non-radical peroxynitrite (ONOO^−^) or S-nitrosoglutathione (GSNO) [12]. The signaling of ROS and RNS are intertwined at various points. The idea of nitro-oxidative stress has become the subject of research in the field of plant biology in the past decade [13]. RNS express their bioactivity through different covalent modifications on specific parts of the proteins. In the process of protein tyrosine nitration ONOO^−^ (product of the reaction of NO and O_2_˙^−^) reacts with tyrosine amino acids in a two-step process, modifying the structure and activity of proteins [14].

HMs are also able to alter the interactions between soil microorganisms [15,16], causing changes in properties of microbial communities such as enzyme activities; thus, providing a valuable tool to assess/understand the effect of HMs on a soil–plant system. Different metals can either increase [17,18] or decrease [19] enzyme functions, proving that microbial dynamics can change differently under HM exposure.

To overcome the challenge of tracking the root growth real time, rhizotrons are widely used tools to in situ observe the development of root system architecture, e.g., in case of trees [20], maize [21], *Arabidopsis* [22], or in our previous study *Brassica napus* under Zn treatment [4]. A common point for all systems used is the transparent wall of the rhizotron, which allows the monitoring of root system with negligible interference.

There are numerous articles dealing with the effect of municipal sewage water and sewage sludge on plant growth and development; however, all of them utilized actual municipal wastewaters, that contain a number of other components in addition to HMs, while the effect of these potentially harmful HMs have not been studied. In several experiments, where urban sewage water is applied, plant growth and functions are usually inhibited, eventually leading to decreased crop yield [23] or accumulation of heavy metals, which could be harmful upon consumption [24].

Hence, our goal was to determine the nitro-oxidative status in the root system of rapeseed (*Brassica napus* L.) and sunflower (*Helianthus annuus* L.) subjected only to combined HM treatment, supplied as an artificially modelled sewage, containing the highest HM concentrations (Cd, Cr, Cu, Hg, Ni, Pb, and Zn) legally allowed.

## 2. Materials and Methods

### 2.1. Rhizotron System, Plant Material, and Growing Conditions

**Figure 1:**
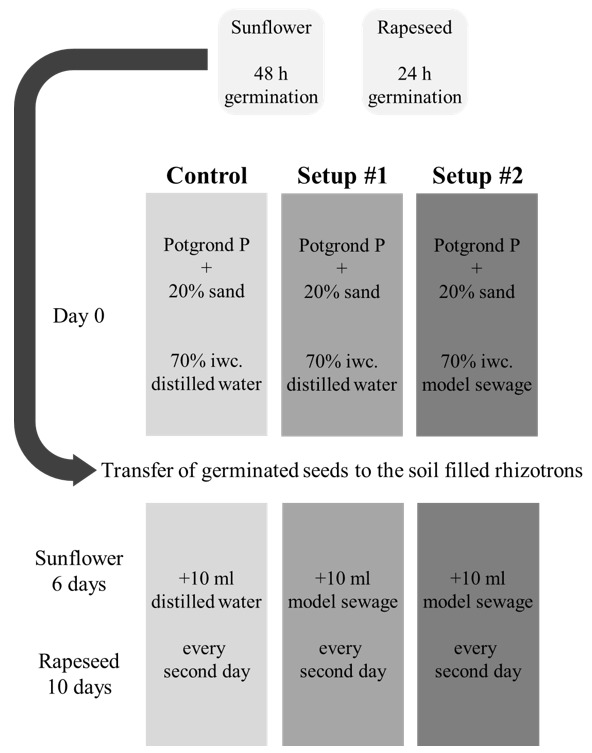
Schematic scheme of the applied growth and treatment system.

The same rhizotron system was utilized as described in [4]. 15 cm wide, 30 cm tall, and 1.6 cm thick rhizotrons were assembled from plexi panels, polifoam sheets, and screws with wing nuts (Figure S1). Rhizotrons were filled with Klasmann Potgrond P (Klasmann-Deilmann GmbH, Geeste, Germany) blocking substrate (100% frozen through black peat with a fine structure of maximum 8 mm size, pH 6.0; 210 mg/L N; 240 mg/L P_2_O_5_, 270 mg/L K_2_O) mixed with 20% sand; the initial water content was set to 70% either by distilled water (control and first-time-sewage-watered systems) or by model sewage (simulating the long term application of sewage water) (Figure 1).

In order to test the effects of combined heavy metal treatment, model sewage water was prepared in accordance with the current Hungarian legalization (Government Regulation 50/2001. (IV.3.)) (Table 1).

Three rhizotron setups were used throughout the experiments. Control rhizotrons were watered with 10 mL distilled water on every second day, while rhizotrons, simulating a soil system irrigated with sewage water for the first time, were watered with 10 mL model sewage water on every second day. Rhizotrons simulating a condition, where sewage was used for irrigation for a longer preceding period, were irrigated with 10 mL model sewage water on every second day, and of which initial water content had been previously set by the model sewage water (Figure 1).

*Brassica napus* L. (GK Gabriella; oil seed rape or rapeseed) and *Helianthus annuus* L. (GK Bambo; sunflower) seeds, provided by the Cereal Research Non-Profit Ltd. (Szeged, Hungary), were pre-germinated for 24 (rapeseed) or 48 (sunflower) h at 26 °C, then germinated seeds were transferred to the soil filled rhizotrons. In the first 48 h, growing seedlings were covered with transparent plastic foil to ensure optimal humidity, then the plants were supplemented with distilled water or model sewage water on every second day as described above. Seedlings were cultivated in greenhouse at photon flux density of 150 µmol/m^2^/s (12/12 h light/dark cycle) at a relative humidity of 55–60% and 25 ± 2 °C for 6 (sunflower) and 10 (rapeseed) days, then all rhizotrons were scanned, disassembled and the roots were cleaned for further examination.

### 2.2. Morphological Measurements

Images acquired by optical scanning were examined using Fiji software (http://fiji.sc/Fiji; [25]). The length of the primary root (PR; cm) was measured; the number of visible lateral roots were counted (LR; laterals per root) and their lengths (mm) and angles included with the primary root (degrees) were also measured.

### 2.3. Element Content Analysis

The concentrations of microelements were measured by inductively-coupled plasma mass spectrometry (ICP-MS, Agilent 7700 Series, Santa Clara, CA, USA) according to [26]. Values of Cd, Cr, Cu, Ni, Pb, and Zn are given in ppb, which equals µg/kg dry weight (DW). Translocation factor ((TF), element concentration in the shoot/element concentration in the root) was calculated according to [27].

### 2.4. Microscopic Determination of Callose and Pectin Deposition and Lipid Peroxidation in the Root Tissues and the Viability of the Root Apical Meristem

Deposition of callose into the cell walls of the root tips was demonstrated by using aniline blue staining according to [28]. Roots tips were incubated in aniline blue solution (0.1%, *w/v* in 1 M glycine) for 5 min, then washed by distilled water prior to microscopic analysis.

Pectin content of the cell walls was detected by using 0.05% (*w/v*) ruthenium red (RR) solution prepared with distilled water, according to [29].

Malondialdehyde, as the product of lipid peroxidation was visualized with Schiff’s reagent after [28].

Viability of meristematic cells in the root tips was determined by fluorescein diacetate (FDA) staining, according to [30]; roots were incubated with 10 µM FDA solution prepared in 10 mM MES (4-morpholineethanesulfonic acid)/50 mM KCl buffer (pH 6.15).

### 2.5. Detection of ROS and RNS

Fluorescence related to superoxide anion in the root tips was detected by using dihydroethidium (DHE) (30 min incubation in darkness at 37 °C with 10 µM dye solution followed by two washing with 10 mM Tris/HCl, pH 7.4) [31]. Hydrogen peroxide-dependent fluorescence was visualized by the incubation of root tips in 50 µM Ampliflu^TM^ (10-acetyl-3,7-dihydroxyphenoxazine, ADHP or Amplex Red) solution (prepared in 50 mM sodium phosphate buffer, pH 7.5) according to [26].

Fluorescence consistent with NO in *Brassica* root tips were demonstrated by 4-amino-5-methylamino-2′,7′-difluorofluorescein diacetate (DAF-FM DA), by incubation in 10 µM dye solution prepared in 10 mM Tris/HCl buffer, (pH 7.4) for 30 min, in darkness, at room temperature [32]. For the in situ and in vivo detection of fluorescence consistent with ONOO^−^ in the root tips, 10 mM 3′-(p-aminophenyl) fluorescein (APF) was applied according to [33].

### 2.6. Acquirement and Processing of Microscopic Images

*Brassica* and *Helianthus* root samples labelled with the fluorescent dyes were studied under a Zeiss Axiovert 200M inverted microscope (Carl Zeiss, Jena, Germany). Filter set 9 (exc.: 450–490 nm, em.: 515–∞ nm) was used for DHE; filter set 10 (exc.: 450–490, em.: 515–565 nm) was applied for DAF-FM, APF and FDA; filter set 20HE (exc.: 546/12, em.: 607/80) was used in case of Amplex Red and filter set 49 (exc.: 365 nm, em.: 445/50 nm) was utilized with aniline blue staining.

Fluorescence intensities (pixel intensity, proportional to the amount of the detected molecule) in the meristematic zone were measured on the images using Axiovision Rel. 4.8 software (Carl Zeiss Microscopy GmbH, Oberkochen, Germany) within circles of 50 µm radii.

### 2.7. Determination of Soil Catalase Activity

Catalase activity in soil was calculated by a titrimetric method according to [34]. Two grams soil from a rhizotron was added to a mixture of 40 mL distilled water and 5 mL 0.3% H_2_O_2_. After 20 min of shaking, 5 mL of 1.5 M H_2_SO_4_ was added and the suspension was filtered, then titrated with 0.02 M KMnO_4_. Catalase activity (CAT) was expressed as µmol H_2_O_2_/g dry soil weight/min calculated from the reacted amount of 0.02 M KMnO_4_. Soil samples without H_2_O_2_ addition were used as blanks.

### 2.8. SDS-PAGE and Western Blotting for Tyrosine Nitration

Protein extracts of *Brassica* and *Helianthus* root tissues were prepared as described in [35]; protein concentration was measured using the Bradford [36] assay with bovine serum albumin as a standard. 20 µL of root protein extracts per lane were subjected to sodium dodecyl sulphate-PAGE (SDS-PAGE) on 12% acrylamide gels, followed by procedures described by [35].

### 2.9. Statistical Analysis

Results are expressed as the mean ± s.e. Multiple comparison analyses were performed with SigmaStat 12 software (Systat Software, Inc., San Jose, CA, USA) using analysis of variance (ANOVA; *p* < 0.05) and Duncan’s test. All experiments were carried out at least two times. In each case at least 10 samples were measured.

## 3. Results and Discussion

### 3.1. Heavy Metal Uptake

**Figure 2:**
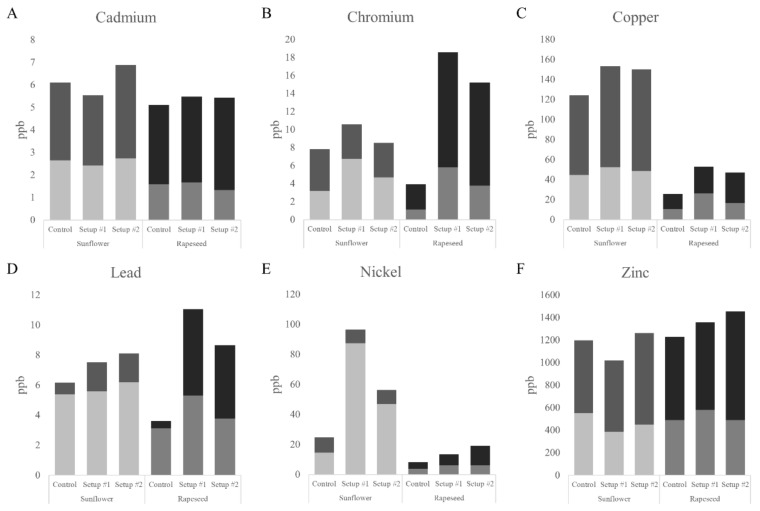
Microelement contents of the roots and shoots of sunflower and rapeseed (ppb): (**A**): cadmium, (**B**): chromium, (**C**): copper, (**D**): lead, (**E**): nickel, and (**F**): zinc. The bottom part of each bar represents the element content of the roots while the top part represents the shoot.

According to the ICP-MS measurements, model-sewage-treatment considerably altered the HM homeostasis in the plants; however, it showed metal- and species-specificity. Despite the applied treatment, Hg content in the plants remained under detection limit. The overall Cd uptake remained generally low in both investigated species, but interestingly both major organs of the sunflower seedlings grown in setup #1 contained less Cd than the controls (Figure 2A). On the other hand, grown in the more severe setup #2, Cd levels of the rapeseed roots decreased compared to that of the control, while eased in their shoots (Figure 2A). Cr levels in both species showed a similar pattern: plants grown on setup #1, where they only encountered combined HM treatment on watering days accumulated more of this metal than plants grown in a constantly and repeatedly contaminated soil (Figure 2B). It is also apparent, that rapeseed was able to accumulate significantly more Cr, than sunflower in both contamination setups. Regarding Cu, sunflower contained it in a generally higher amount than rapeseed, while it is true for both species that plants’ roots grown in setup #1 accumulated a higher amount of Cu than those grown in setup #2 (Figure 2C). Pb levels in both organs of sunflower also increased with the increasing pollution level of the model sewage. While in rapeseed, Pb content was significantly higher in plants grown in setup #1 than in the more polluted setup #2 (Figure 2D). Ni content of sunflower followed the same pattern as Cu and Cr; however, in rapeseed the generally lower Ni levels increased in a contamination-severity-dependent manner (Figure 2E). Zn content of sunflower changed similarly to Cd levels: the seedlings grown in setup #1 contained less Zn, than the controls (Figure 2F). In the case of rapeseed, the total amount of Zn increased with the severity of the model HM pollution, while the Zn content of the plant’s root system increased only in setup #1 (Figure 2F).

In general, combined HM treatment altered the balance of microelement homeostasis of both species. Interestingly, in the case of sunflower the less severe HM treatment in setup #1 caused higher total accumulation of Cr, Cu, and Ni than the more polluted setup #2. In rapeseed, such a response occurred as well, but in the accumulated amount of Cd, Cr, Cu, and Pb, showing that different metals were accumulated by different plant species under the same circumstances. Plants are able to modify their cell wall composition to either indicate or prevent heavy metal uptake. Callose and pectin content in the root tips of sunflower increased slightly in both setups, especially in setup #1 (Figure S2A,C), raising the possibility of the correlation between the highest measured Cr, Cu, and Ni accumulation in the root and callose deposition, as a partially failed attempt to exclude these HMs, since callose would be impermeable to metal ions [37]. The highest increase in pectin content could also support the high metal content, since pectin is able to bind heavy metals in the cell walls [38]; however, this does not seem to be generally true to all examined HMs.

In case of rapeseed, callose content decreased in root tips grown in setup #1 in parallel to the highest measured Cd, Cr, Cu, Pb, and Zn accumulation in the roots (Figure S2B), while pectin content increased in both setups, especially in setup #1 (Figure S2D). The change in callose deposition is just the opposite compared to sunflower, suggesting that rapeseed is more tolerant to HMs and also more capable to take them up in a scenario modelled by setup #1. The high pectin content of the root tips might play a role in the high measured HM content in this case, binding them to the cell walls, similar to previously hypothesized in the case of sunflower.

### 3.2. Translocation of HMs

**Figure 3:**
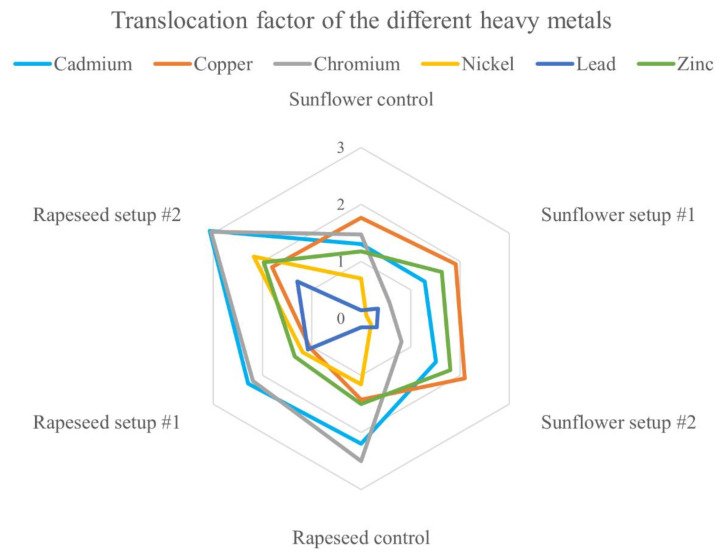
Translocation factor (element concentration in the shoot/element concentration in the root) of the investigated heavy metals in the two species grown in different setups.

Translocation factor (TF) is calculated as a shoot to root concentration ratio and is suitable for assessing the phytoremediation potential of plants [39]. Since TF values remained under one, sunflower seems to keep Cr, Ni, and Pb in its root system after model-sewage treatment, while in case of Cd, Cu, and Zn, t showed a moderate translocation capacity (TF value only reaching two in case of Cu in setup #2). On the contrary, rapeseed presented a great translocation capacity in case of Cd and Cr (TF values exceeding two and three), while in case of setup #2, Zn and Ni TF values also reached two, suggesting that rapeseed is more capable of translocating heavy metals to above-ground parts, proposing that rapeseed might be a better candidate for phytoremediation processes (Figure 3).

Previous studies have shown that though plants might tolerate treatment with sewage water, their growth and development can be inhibited and also might impose a health risk upon accumulating a large amount of heavy metals [40,41,42,43,44]. Upon irrigation with sewage water, rapeseed has been shown to take up and translocate Cd and Cr to its shoot in proportion with the level of sewage water treatment [45]. Sunflower treated with sewage sludge also showed increased concentration of Cr, Cu, Ni, and Zn compared to control plants [46].

### 3.3. Changes in Root System Architecture

Changes in the element availability, uptake, and homeostasis by the different application of the model sewage affected the root growth and development of the two species differently.

**Figure 4:**
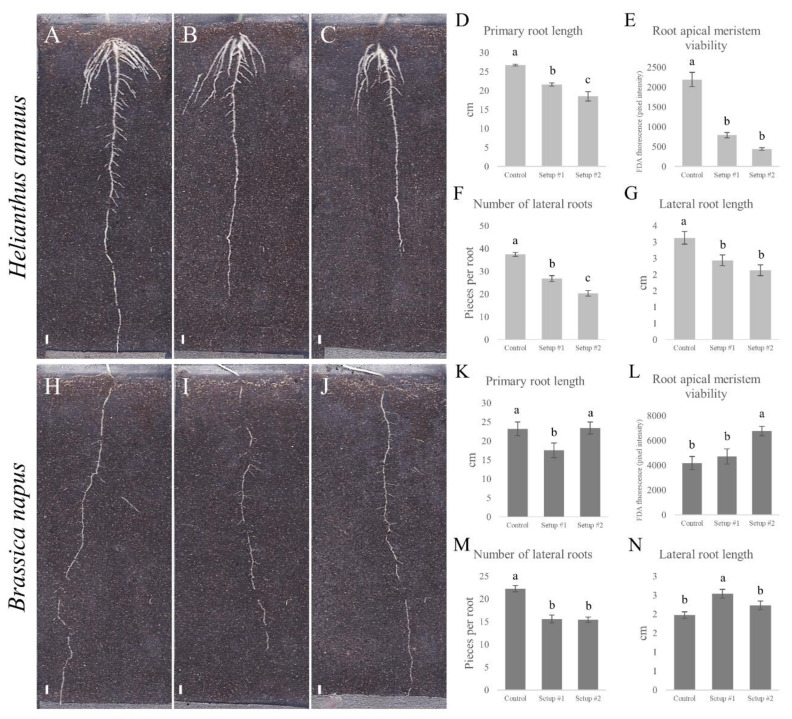
(**A**–**C**), (**H**–**J**) Representative images of the effect of model sewage on the root system architecture of 6-day-old *H. annuus* and 10-day-old *B. napus*. ((**A**/**H**): control, (**B**/**I**): Setup #1, and (**C**/**J**): Setup #2; bar = 1 cm). Length of the primary root ((**D**) sunflower, (**K**) rapeseed); viability of the root apical meristem ((**E**) sunflower, (**L**) rapeseed); and number ((**F**) sunflower, (**M**) rapeseed) and length of lateral roots ((**G**) sunflower, (**N**) rapeseed). Different letters indicate significant differences according to Duncan-test (*p* < 0.05).

Both treatment setups inhibited root growth of sunflower, proportional to the severity of the model sewage treatment (Figure 4A–C). Primary root length decreased significantly in both setups (by 20 and 31% in setup #1 and setup #2, respectively) (Figure 4D), which can be explained by the significant decrease (by 64 and 80% in setup #1 and setup #2, respectively) in the metabolic activity and viability of the root apical meristems (Figure 4E). In addition to the longitudinal growth, lateral expansion of the root system was also inhibited by the combined HM treatments, since both the number (Figure 4E; by 27 and 46% in setup #1 and setup #2, respectively) and length (Figure 4G; by 22 and 32% in setup #1 and setup #2, respectively) of lateral roots decreased significantly. In addition to the length of the lateral roots, their angle included with the primary root changed significantly due to combined HM treatment (Figure S3A). From the 62° measured under control circumstances, angles increased to 67° in setup #1 and to 69° in the more severe setup #2, which means that the intensifying combined HM treatment resulted in a more horizontal orientation of the lateral roots compared to the control conditions.

On the other hand, rapeseed responded differently to the model sewage treatment, since root growth decreased only in setup #1 (Figure 4H–J), which was confirmed by the primary root length data (Figure 4K), where it decreased by 26% in setup #1. In the background of the relative model sewage-tolerance, increased metabolic activity and viability of the apical meristem was found (Figure 4L; by 26 and 61%). Lateral root expansion was also affected by combined HM treatment. The number of lateral roots decreased in both setups by 32% (Figure 4M), while the length of the lateral roots increased significantly (by 28%) in setup #1 and did not change in setup #2. Orientation of the lateral roots also changed to be more horizontal (from the control 74° to 76° and 80° in setup #1 and setup #2, respectively). Although the direction of change was the same, it was slightly less pronounced than in sunflower and only significant in setup #2 (Figure S3B). The physiological explanation behind HM-induced changes of the angle of lateral roots to primary root is mostly still unknown. In our previous work, Zn in high concentration caused the lateral roots to grow in a more vertical direction while growth-promoting Zn supplementation induced a more horizontal lateral root alignment [4]. In the present case, based on the above-mentioned observations, the changes in lateral root angles indicate that rapeseed is tolerant to the applied combined HM treatments. However, it has to be noted that while the growth of the lateral roots of sunflower also changed to the horizontal direction, combined HM treatments posed a stress and inhibited root growth, based on all the other root system architecture parameters, so the use of lateral root angles as an indicator of stress or tolerance seems to show a species-specificity.

According to the changes in root growth and architecture under the combined HM treatment with different severity, it can be concluded that rapeseed is in general more tolerant to the presence of multiple HMs, based on its ability to maintain better root growth compared to sunflower. As it was presented before, rapeseed showed a generally higher translocation capacity of the investigated heavy metals compared to sunflower, which might be partly responsible for its better relative tolerance to these potentially toxic elements in terms of root growth.

### 3.4. Changes in the Nitro-Oxidative Status

**Figure 5:**
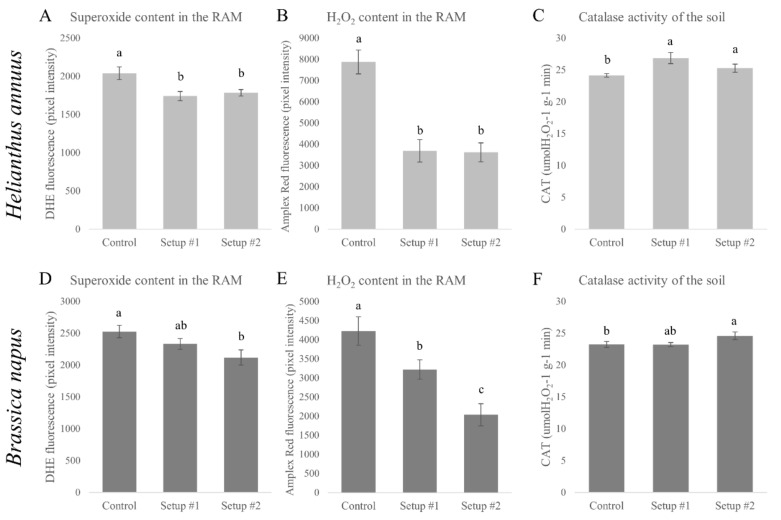
Fluorescence representing superoxide content in the root of *H. annuus* (**A**) and *B. napus* (**D**) in different setups. Hydrogen peroxide linked fluorescence in the roots of *H. annuus* (**B**) and *B. napus* (**E**) in different setups. Soil catalase activity in the different setups after 6 days with the presence of sunflower (**C**) or 10 days with the presence of rapeseed (**F**). Different letters indicate significant differences according to Duncan-test (*p* < 0.05).

Fluorescence, correlating with ROS and RNS content of the root tips, were measured in both species and setups. In the case of sunflower, both investigated ROS, including O_2_˙^−^ (Figure 5A) and H_2_O_2_ (Figure 5B) content, decreased significantly and equally in all two-model sewage-treated setups compared to the control. Catalase activity in soil was also measured, since it seemed to be correlated with the H_2_O_2_ content of the root tips in a previous study [4]. In the present experiments, soil catalase activity significantly increased in both sewage-treated rhizotron systems, when sunflower was grown in them (Figure 5C). This observation raises the possibility that catalase activity in soil might contribute to the decreased H_2_O_2_ content of the root tips. Despite the increased soil catalase activity and decreased amount of ROS, the appearance of the products of lipid peroxidation was detected in both setups (Figure S4A). Normally, lipid peroxidation indicates oxidative damage under stress [47], but according to our ROS data this was not the case; thus, the possible activation of antioxidant defense systems also cannot be ruled out.

In case of rapeseed, fluorescence, consistent with O_2_˙^−^ content, also decreased by combined HM treatments, depending on the severity of treatments (Figure 5D). H_2_O_2_-linked fluorescence showed a severity-dependent decrease compared to the control (Figure 5E), while in parallel, soil catalase activity increased slightly in setup #1 and significantly in setup #2 (Figure 5F). In contrast with sunflower, in rapeseed root tips no lipid peroxidation could be detected (Figure S4B) in any of the setups.

**Figure 6:**
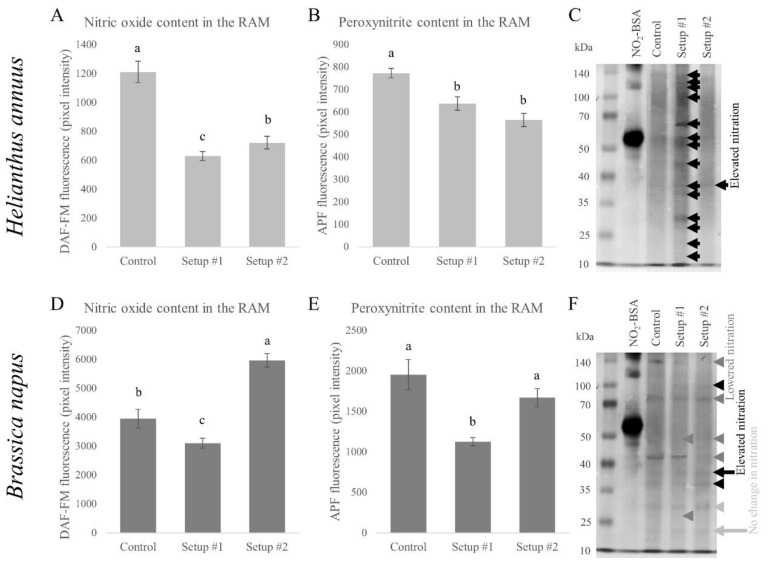
Changes in the fluorescence consistent with nitric oxide in sunflower (**A**) and rapeseed (**D**) root tips. Fluorescence proportional to peroxynitrite content in sunflower (**B**) and rapeseed (**E**) root tips. Different letters indicate significant differences according to Duncan-test (*p* < 0.05). Representative immunoblot showing protein tyrosine nitration in the roots of sunflower (**C**) and rapeseed (**F**) grown in different rhizotron setups. Black arrows show elevated nitration; dark grey arrows show lowered nitration compared to the control. Light grey arrows indicate protein bands with not altered nitration due to the model sewage treatment.

While changes in ROS (O_2_˙^−^ and H_2_O_2_) levels showed a similar, decreasing tendency in the root tips of both species, combined HM treatment influenced the nitrosative homeostasis quite differently. In sunflower, fluorescence dependent on NO and ONOO^−^ content of the root tips decreased significantly in both setups (Figure 6A,B). Interestingly, the decreased RNS level is coupled with a significantly increased protein tyrosine nitration in setup #1, while in setup #2, strength of nitration was more similar to the control’s (Figure 6C). In rapeseed, fluorescence, consistent with NO and ONOO^−^, decreased significantly by the less severe combined HM treatment compared to the control (Figure 6D,E), and this was accompanied with decreased protein tyrosine nitration (Figure 6F). On the other hand, the more severe model sewage treatment caused a significant nitric oxide accumulation (Figure 6D), which (together with a slightly decreased peroxynitrite content) caused the reorganization of nitration pattern; in some cases, nitration increased, decreased, or did not change (Figure 6F).

Protein tyrosine nitration is a ONOO^−^-dependent process [14]; however, this does not necessarily mean that changes in their level can be detected simultaneously due to the various intermolecular processes that might happen in the cells. The existence of the physiological nitroproteome has been known for more than a decade now [33], and it has been shown in numerous species (reviewed by [48]). Like many other abiotic stressors (reviewed by [49]), HMs are able to disturb the nitro-oxidative homeostasis of the plants and induce changes in protein tyrosine nitration. It was shown previously, that, for example, Cd was able to increase nitration in soybean roots [50], Zn in *Brassica* species [28,51], and Ni in *Arabidopsis thaliana* and *Brassica juncea* [6]. It has also been shown that different degrees of Zn exposure causes different responses in the level and pattern of nitration; Zn in a low, growth inducing concentration caused a change in nitration pattern, while a high, stress inducing amount of Zn generally increased nitration in the roots [4]. Based on the gathered knowledge so far, the significantly increased nitration of sunflower root in setup #1 indicates a severe stress state. The control-like nitration in the more severe setup #2 could be explained by strong growth inhibition and the loss of viability, where protein content can be decreased by excess stress in order to reverse injury [52]. In rapeseed no general increase in protein tyrosine nitration could be detected, since in setup #1 the intensity of some nitrated bands decreased; while in setup #2 both increased and decreased bands could be detected, which was accompanied by significant increase in NO content of the root tips. These results support the previously obtained data with Zn [4], that tolerance to HMs can be indicated by an altered pattern of nitration, and it seems to apply to scenarios when more than one HM is involved.

## 4. Conclusions

The present study compared the effect of two combined heavy metal treatments (modelling municipal sewage) with different severity on the early root growth and development together with the underlying processes in sunflower and rapeseed, grown in a soil-filled rhizotron system. Our novel experimental design applied in this study is unique, since it focused on assessing the effect of multiple heavy metals without any additional possible stressors or modifying components commonly found in municipal sewages.

Both species reacted differently to the combined HM stress on several levels. Compared to rapeseed, sunflower proved to be relatively sensitive to combined HM treatment according to root growth parameters, accompanied by a lower metal translocation capability, decreased ROS and RNS content of the root tips, and a significantly intensified protein tyrosine nitration in the less severe combined HM treatment.

In the more tolerant rapeseed, metal translocation capability was generally higher. Growth inhibition by the less severe combined HM treatment, in terms of primary root length and lateral root number, is coupled with decreased nitric oxide and peroxynitrite levels compared to the control, resulting in a generally decreased protein tyrosine nitration. Interestingly, by the more severe combined HM treatment, only lateral root number decreased compared to the control, while the significantly increased viability of the root tips was accompanied by increased nitric oxide content and reorganized protein tyrosine nitration pattern. This ultimately resulted in a reorganized nitration pattern, which could be considered as a marker of tolerance against combined heavy metal treatment.

The obtained results suggest that, in case of rapeseed, an increased nitric oxide content and changed pattern of tyrosine nitration could indicate acclimation to combined heavy metal treatment.

## Figures and Tables

**Table 1:** Limit values for toxic elements and harmful substances in waste-water for agricultural use according to the current Hungarian legalization (Government Regulation 50/2001. (IV.3.)).

Heavy Metal	Allowed Amount (ppm)
Cadmium (Cd)	0.02
Copper (Cu)	2.0
Chromium (Cr)	0.5
Mercury (Hg)	0.01
Nickel (Ni)	1.0
Lead (Pb)	1.0
Zinc (Zn)	5.0

## Supplementary Materials

The following are available online at https://www.mdpi.com/2223-7747/9/7/902/s1, Table S1: Properties and effects of cadmium, chromium, copper, lead, mercury, nickel, and zinc on plants., Figure S1: Schematic design of the rhizotron system applied (A) and growing *B. napus* seedlings in the rhizotrons filled with soil (B)., Figure S2: Callose (A) and pectin (C) content in the root tip of 10 day-old *H. annuus* and 6 day-old *B. napus* (B,D) grown in control soil or in soil differently treated with combined heavy metals., Figure S3: Visualization of the changes in the angle of the lateral roots with the primary root., Figure: S4. Representative images of Schiff staining in order to detect lipid peroxidation in sunflower (A) and rapeseed (B) root tips under control circumstances and submitted to different combined heavy metal treatment.
