# Supplementary material for: Reorganization of Protein Tyrosine Nitration Pattern Indicates the Relative Tolerance of Brassica napus (L.) over Helianthus annuus (L.) to Combined Heavy Metal Treatment
Source: Plants (Basel). 2020 Jul 16;9(7):902. doi: 10.3390/plants9070902 (PMC7411833; doi:10.3390/plants9070902)
Supplement: Supplementary file 1 [file plants-09-00902-s001.pdf]

Supplementary material

Table S1. Properties and effects of cadmium, chromium, copper, lead, mercury, nickel and zinc on plants.

Metal	Essential in plants	Mobility in plants	Accumulates in	Effects in excess	References
Cadmium	No	Mobile	All in their organs	Growth inhibition.	S1-S3
Chromium	No	Low	More in roots	Growth inhibition, oxidative stress.	S4-S8
Copper	Yes	Mobile	All in their organs	Growth inhibition, chlorosis and necrosis.	S9-S11
Lead	No	Low	Roots	Imbalance in the microelement homeostasis, inhibition of growth and photosynthesis.	S12-S16
Mercury	No	Low	Roots	Growth inhibition, negative effect on metabolism, photosynthesis, nutrient uptake, antioxidant enzyme capacity.	S17-S20
Nickel	Yes	Mobile	More in the aerial parts	Inhibition of photosynthesis, nitrogen assimilation. DNA damage.	S21-S26
Zinc	Yes	Mobile	All in their organs	Phytotoxicity	S27-S30

Cadmium is a mayor contaminant in the environment. According to our knowledge, it is the only HM which can be accumulated in such a concentration which is not toxic to plants but poses health risks both to animals and humans upon entering the food chain by bioaccumulation [S1, S2]. Cd is a mobile element in soil, plants can take up, transport and distribute it to all of their organs effectively [S3].

Chromium is a naturally occurring metal in volcanic dust and rocky soils [S4], easily contaminating groundwater due to its high solubility. Although, in appropriate quantities, Cr is an essential nutrient for the human body, it has no biological role in plant physiology [S5]. Plants do not have any specific uptake mechanism for Cr [S6], but conversion of Cr into organic complexes in the presence of acidic root exudates increase its accumulation [S7]. In comparison with other HMs Cr shows higher accumulation in roots due to its low mobility and its sequestration into root cell vacuoles [S8].

As an essential micronutrient, copper is necessary for the optimal growth and development of plants, but in excess, it becomes toxic. Cu is easily accessible for plants directly from the soil solution as free cations or by chelation [S9], however, it has a low diffusion capability in soil solution, hence its availability might be limited [S10]. Plants exposed to moderate amount of excess Cu show stress-induced morphogenic response (SIMR) [S11], while in high quantity, toxicity symptoms such as growth inhibition, chlorosis and necrosis occur [S9].

Lead, after being adsorbed by soil particles, may occur in numerous chemical forms with a various level of mobility, availability and toxicity, but in general, only a fraction of Pb is available for plants in a soluble form due to its high affinity to bind to organic and colloidal materials [S12]. Moreover, Cd, Cr, Cu, Ni and Zn negatively affect the availability of Pb for plants, further restricting its accessibility [S13]. If taken up, similarly to Cr, Pb is primarily accumulated in roots with a restricted translocation to the shoot [S14, S15]. Accumulation of Pb in plants, in one hand, disturbs their nutrient uptake, growth and photosynthetic processes [S12], on the other hand, it also poses a health risk to humans by entering the food chain [S16].

Although mercury is highly soluble in water, its uptake from soil can be decelerated by high soil pH or high amount of salts and lime [S17]. Hg, often bound with sulphur and nitrogen ligands, enters animal cells via ionic channels competing with Cu, Fe or Zn [S18]. Nevertheless, the exact mechanism in plants is still yet to be discovered. Adsorbed Hg remains primarily in the roots [S19]; in excess, it inhibits plant growth by its negative effects on metabolism, photosynthesis, nutrient uptake and antioxidant enzyme capacity [S17, S20].

Nickel was classified as an essential element for plant growth only in 1987 [S21] and is solely known to have a biochemical function in the active site of urease enzyme [S22]. Due to its limited necessity, plants require and contain only a low amount of Ni [S23]. In excess, it inhibits photosynthesis, nitrogen assimilation and various enzyme activities, while damaging DNA [S24]. Availability on Ni for plants depends on soil structure, free iron (Fe) and manganese (Mn) oxides, soil organic matter and pH [S25]. Since the proof of existence for Ni-specific transporters is lacking in plants, non-selective transporters are responsible for Ni uptake by roots [S26].

Zinc is the second most common metal in organisms [S27], with diverse functions in life processes [S28]. A typical agricultural soil contains 10-300 μg/g Zn, however, anthropogenic activities such as mining, excessive use of fertilisers or application of sewage can increase its amount [S29, S30]. In excess, Zn causes phytotoxicity, but the exact effect depends on the dose and plant species examined.

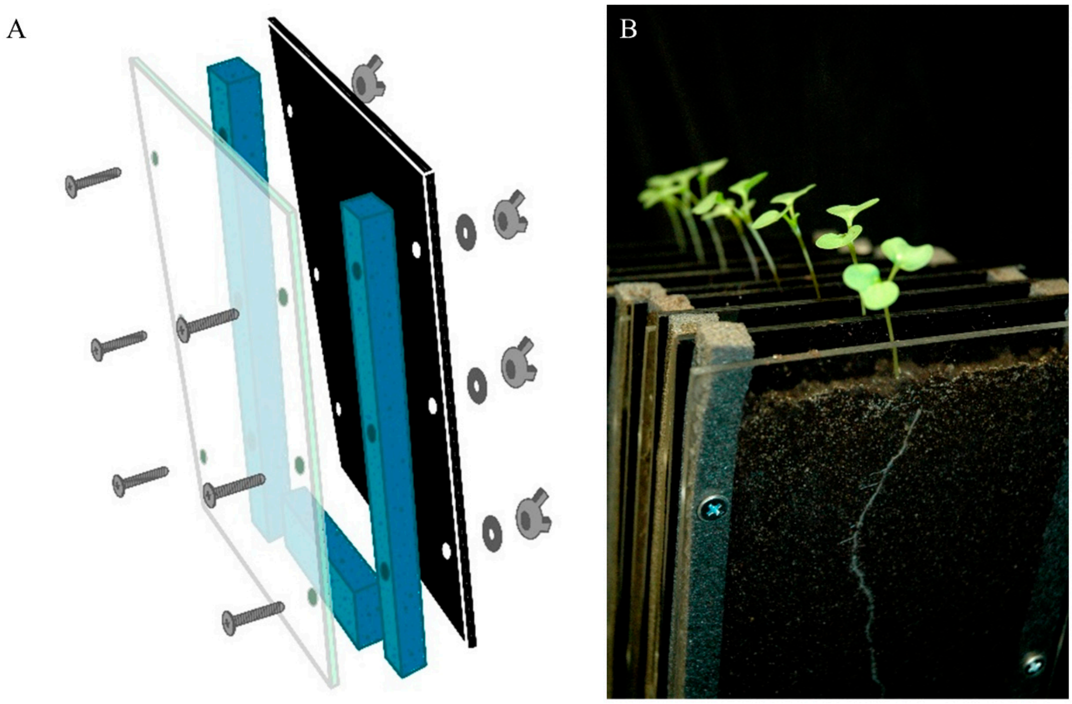

Figure S1. Schematic design of the rhizotron system applied (A) and growing *B. napus* seedlings in the rhizotrons filled with soil (B) (adapted from [4]).

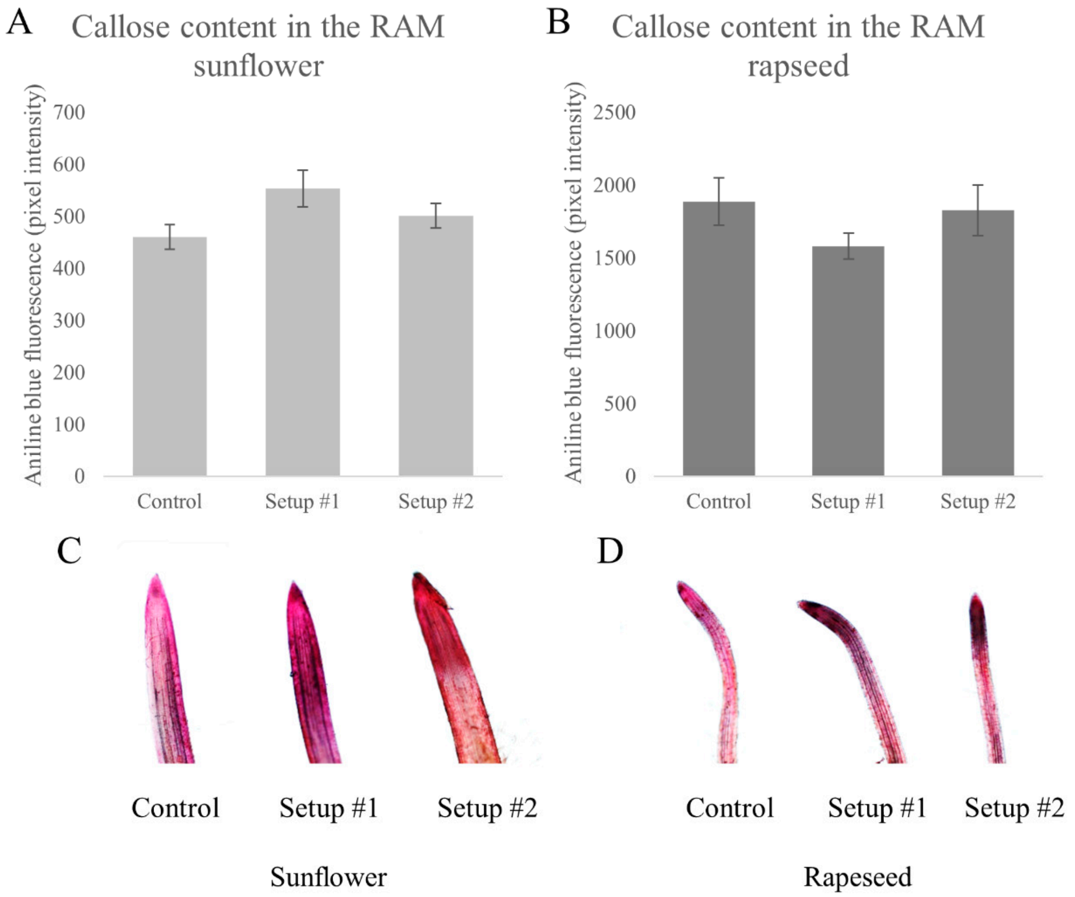

Figure S2. Callose (A) and pectin (C) content in the root tip of 10 days-old *H. annuus* and 6 days-old *B. napus* (B and D) grown in control soil or in soil differently treated with combined heavy metals.

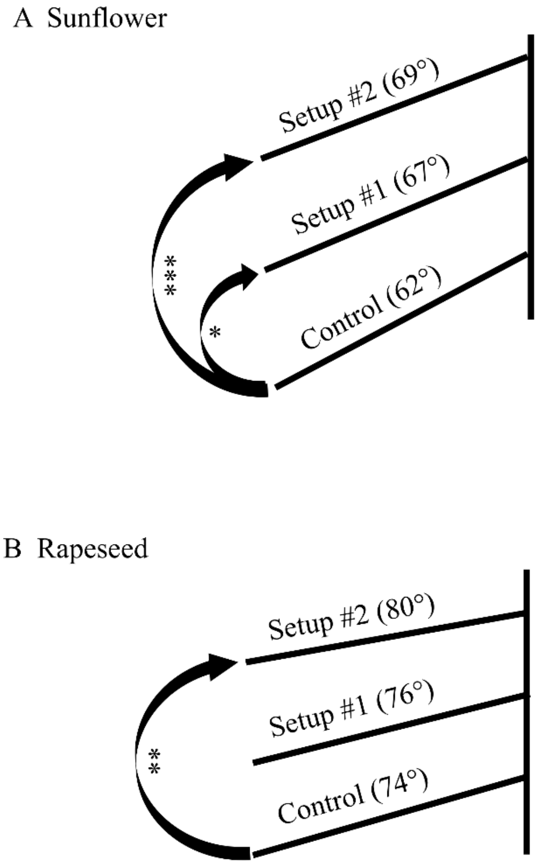

Figure S3. Visualisation of the changes in the angle of the lateral roots with the primary root. In case of sunflower, from the 62°measured under control circumstances, angles increased to 67% in setup #1 and 69° in the more severe setup #2 (A), while orientation of the rapeseed lateral roots also changed to be more horizontal (from the control 74° to 76° and 80° in setup #1 and 2, respectively), however it was less pronounced than in sunflower and only significant in setup #2.

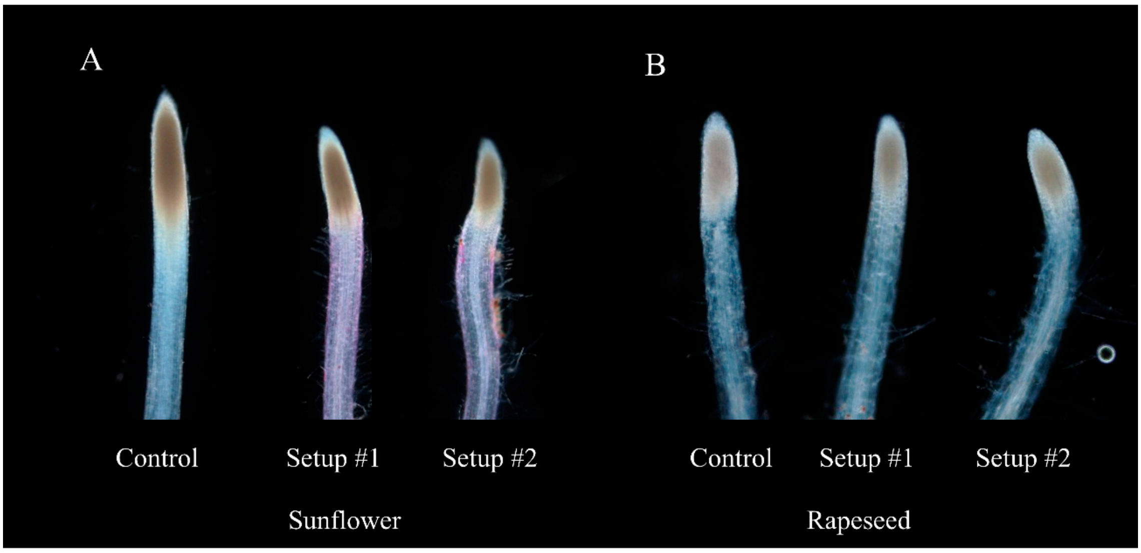

Figure S4. Representative images of Schiff staining in order to detect lipid peroxidation in sunflower (A) and rapeseed (B) root tips under control circumstances and submitted to different combined heavy metal treatment. Pink coloration indicates peroxidation of membrane lipids.

Supplementary references

1. Peijnenburg, W.; Baerselman, R.; de Groot, A.; Jager, T.; Leenders, D.; Posthuma, L.; Van Veen, R. Quantification of Metal Bioavailability for Lettuce (Lactuca sativa L.) in Field Soils. *Archives of Environmental Contamination and Toxicology* **2000**, *39*, 420–430; DOI:10.1007/s002440010123
2. Ismael, M.; Elyamine, A.; Moussa, M.; Cai, M.; Zhao, X.; Hu, C. Cadmium in plants: uptake, toxicity, and its interactions with selenium fertilizers. *Metallomics* **2019**, *11*, 255–277; DOI:10.1039/C8MT00247A
3. Gjorgieva Ackova, D. Heavy metals and their general toxicity for plants. *Plant Science Today* **2018**, *5*, 14; DOI:10.14719/pst.2018.5.1.355
4. Sharma, A.; Kapoor, D.; Wang, J.; Shahzad, B.; Kumar, V.; Bali, A.; Jasrotia, S.; Zheng, B.; Yuan, H.; Yan, D. Chromium Bioaccumulation and Its Impacts on Plants: An Overview. *Plants* **2020**, *9*, 100; DOI:10.3390/plants9010100
5. Reale, L.; Ferranti, F.; Mantilacci, S.; Corboli, M.; Aversa, S.; Landucci, F.; Baldisserotto, C.; Ferroni, L.; Pancaldi, S.; Venanzoni, R. Cyto-histological and morpho-physiological responses of common duckweed (Lemna minor L.) to chromium. *Chemosphere* **2016**, *145*, 98–105; DOI:10.1016/j.chemosphere.2015.11.047
6. Oliveira, H. Chromium as an Environmental Pollutant: Insights on Induced Plant Toxicity. *Journal of Botany* **2012**, *2012*, 1–8; DOI:10.1155/2012/375843
7. Srivastava, S.; Srivastava, S.; Prakash, S.; Srivastava, M. Fate of trivalent chromium in presence of organic acids: a hydroponic study on the tomato plant. *Chemical Speciation & Bioavailability* **1998**, *10*, 147–150; DOI:10.3184/095422998782775763
8. Mangabeira, P.; Ferreira, A.; de Almeida, A.; Fernandes, V.; Lucena, E.; Souza, V.; dos Santos Júnior, A.; Oliveira, A.; Grenier-Loustalot, M.; Barbier, F.; Silva, D. Compartmentalization and ultrastructural alterations induced by chromium in aquatic macrophytes. *BioMetals* **2011**, *24*, 1017–1026; DOI:10.1007/s10534-011-9459-9
9. Marschner, H. Marschner's mineral nutrition of higher plants. Academic press, 2011; DOI:10.1016/C2009-0-63043-9
10. Gupta, U.C. Copper in agricultural crops. In: Nriagu, J.O. (Ed.), Copper in the Environment. Wiley, New York, 1979, pp. 255–287.
11. Potters, G.; Pasternak, T.; Guisez, Y.; Palme, K.; Jansen, M. Stress-induced morphogenic responses: growing out of trouble?. *Trends in Plant Science* **2007**, *12*, 98–105; DOI:10.1016/j.tplants.2007.01.004
12. Mitra, A.; Chatterjee, S.; Voronina, A.; Walther, C.; Gupta, D. Lead Toxicity in Plants: A Review. *Radionuclides and Heavy Metals in the Environment* 2019, 99–116; DOI:10.1007/978-3-030-21638-2_6
13. Orroño, D.; Schindler, V.; Lavado, R. HEAVY METAL AVAILABILITY INPELARGONIUM HORTORUMRHIZOSPHERE: INTERACTIONS, UPTAKE AND PLANT ACCUMULATION. *Journal of Plant Nutrition* **2012**, *35*, 1374–1386; DOI:10.1080/01904167.2012.684129
14. LANE, S.; MARTIN, E. A HISTOCHEMICAL INVESTIGATION OF LEAD UPTAKE IN RAPHANUS SATIVUS. *New Phytologist* **1977**, *79*, 281–286; DOI:10.1111/j.1469-8137.1977.tb02206.x
15. Kumar, P.; Dushenkov, V.; Motto, H.; Raskin, I. Phytoextraction: The Use of Plants To Remove Heavy Metals from Soils. *Environmental Science & Technology* **1995**, *29*, 1232-1238; DOI:10.1021/es00005a014
16. Ashraf, U.; Kanu, A.; Deng, Q.; Mo, Z.; Pan, S.; Tian, H.; Tang, X. Lead (Pb) Toxicity; Physio-Biochemical Mechanisms, Grain Yield, Quality, and Pb Distribution Proportions in Scented Rice. *Frontiers in Plant Science* 2017, *8*; DOI:10.3389/fpls.2017.00259
17. Azevedo, R.; Rodriguez, E. Phytotoxicity of Mercury in Plants: A Review. *Journal of Botany* **2012**, *2012*, 1–6; DOI:10.1155/2012/848614
18. Blazka, M.; Shaikh, Z. Cadmium and mercury accumulation in rat hepatocytes: Interactions with other metal ions. *Toxicology and Applied Pharmacology* **1992**, *113*, 118–125; DOI:10.1016/0041-008X(92)90015-K
19. Lenka, M.; Panda, K.; Panda, B. Monitoring and assessment of mercury pollution in the vicinity of a chloralkali plant. IV. Bioconcentration of mercury in in situ aquatic and terrestrial plants at Ganjam, India. *Archives of Environmental Contamination and Toxicology* **1992**, *22*, 195–202; DOI:10.1007/BF00213285
20. Calgaroto, N.; Castro, G.; Cargnelutti, D.; Pereira, L.; Gonçalves, J.; Rossato, L.; Antes, F.; Dressler, V.; Flores, E.; Schetinger, M.; Nicoloso, F. Antioxidant system activation by mercury in Pfaffia glomerata plantlets. *BioMetals* **2010**, *23*, 295–305; DOI:10.1007/s10534-009-9287-3
21. Brown, P.; Welch, R.; Cary, E. Nickel: A Micronutrient Essential for Higher Plants. *Plant Physiology* **1987**, *85*, 801–803; DOI:10.1104/pp.85.3.801

22. Balasubramanian, A.; Ponnuraj, K. Crystal Structure of the First Plant Urease from Jack Bean: 83 Years of Journey from Its First Crystal to Molecular Structure. *Journal of Molecular Biology* **2010**, *400*, 274–283; DOI:10.1016/j.jmb.2010.05.009
23. Chen, C.; Huang, D.; Liu, J. Functions and Toxicity of Nickel in Plants: Recent Advances and Future Prospects. *CLEAN - Soil, Air, Water* **2009**, *37*, 304–313; DOI:10.1002/clen.200800199
24. Yusuf, M.; Fariduddin, Q.; Hayat, S.; Ahmad, A. Nickel: An Overview of Uptake, Essentiality and Toxicity in Plants. *Bulletin of Environmental Contamination and Toxicology* **2010**, *86*, 1–17; DOI:10.1007/s00128-010-0171-1
25. Shahzad, B.; Tanveer, M.; Rehman, A.; Cheema, S.; Fahad, S.; Rehman, S.; Sharma, A. Nickel; whether toxic or essential for plants and environment - A review. *Plant Physiology and Biochemistry* **2018**, *132*, 641–651; DOI:10.1016/j.plaphy.2018.10.014
26. Van der Pas, L.; Ingle, R. Towards an Understanding of the Molecular Basis of Nickel Hyperaccumulation in Plants. *Plants* **2019**, *8*, 11; DOI:10.3390/plants8010011
27. Andreini, C.; Bertini, I.; Rosato, A. Metalloproteomes: A Bioinformatic Approach. *Accounts of Chemical Research* **2009**, *42*, 1471–1479; DOI:10.1021/ar900015x
28. Broadley, M.; White, P.; Hammond, J.; Zelko, I.; Lux, A. Zinc in plants. *New Phytologist* **2007**, *173*, 677–702; DOI:10.1111/j.1469-8137.2007.01996.x
29. Bacon, J.; Dinev, N. Isotopic characterisation of lead in contaminated soils from the vicinity of a non-ferrous metal smelter near Plovdiv, Bulgaria. *Environmental Pollution* **2005**, *134*, 247–255; DOI:10.1016/j.envpol.2004.07.030
30. Bi, X.; Feng, X.; Yang, Y.; Qiu, G.; Li, G.; Li, F.; Liu, T.; Fu, Z.; Jin, Z. Environmental contamination of heavy metals from zinc smelting areas in Hezhang County, western Guizhou, China. *Environment International* **2006**, *32*, 883–890; DOI:10.1016/j.envint.2006.05.010
